# Elevated urine BMP phospholipids in LRRK2 and VPS35 mutation carriers with and without Parkinson’s disease

**DOI:** 10.1038/s41531-023-00482-4

**Published:** 2023-04-04

**Authors:** Sara Gomes, Alicia Garrido, Francesca Tonelli, Donina Obiang, Eduardo Tolosa, Maria José Martí, Javier Ruiz-Martínez, Ana Vinagre-Aragón, Haizea Hernandez-Eguiazu, Ioana Croitoru, Vicky L. Marshall, Theresa Koenig, Christoph Hotzy, Frank Hsieh, Marianna Sakalosh, Elizabeth Tengstrand, Shalini Padmanabhan, Kalpana Merchant, Christof Bruecke, Walter Pirker, Alexander Zimprich, Esther Sammler

**Affiliations:** 1 Medical Research Council Protein Phosphorylation and Ubiquitylation Unit, University of Dundee, Dundee, DD1 5EH UK; 2 Parkinson’s Disease and Movement Disorders Unit, Institut Clínic de Neurociències, Hospital Clinic Universitari, Barcelona, Spain; 3 Centre for Networked Biomedical Research on Neurodegenerative Diseases (CIBERNED), Madrid, Spain; 4 Department of Clinical and Experimental Neurology, Laboratory of Parkinson disease and other Neurodegenerative Movement Disorders (IDIBAPS), University of Barcelona, Barcelona, Spain; 5 Hospital Universitario Donostia, San Sebastián, Spain; 6 Group of Neurodegenerative Diseases, Biodonostia Research Institute, San Sebastián, Spain; 7 Neurology, Queen Elizabeth University Hospital, Institute of Neurological Sciences, Glasgow, UK; 8 Department of Neurology, Medical University of Vienna, Wien, Austria; 9 Nextcea, Inc. 500 West Cummings Park, Suite 4550, Woburn, MA USA; 10 The Michael J. Fox Foundation for Parkinson’s Research, New York, NY USA; 11 Northwestern University Feinberg School of Medicine, Chicago, IL USA; 12 Department of Neurology, Klinik Ottakring, Vienna, Austria; 13 Molecular and Clinical Medicine, Ninewells Hospital and Medical School, University of Dundee, Dundee, UK

**Keywords:** Parkinson's disease, Parkinson's disease, Translational research

## Abstract

Elevated urine bis(monoacylglycerol)phosphate (BMP) levels have been found in gain-of-kinase function LRRK2 G2019S mutation carriers. Here, we have expanded urine BMP analysis to other Parkinson’s disease (PD) associated mutations and found them to be consistently elevated in carriers of LRRK2 G2019S and R1441G/C as well as VPS35 D620N mutations. Urine BMP levels are promising biomarkers for patient stratification and potentially target engagement in clinical trials of emerging targeted PD therapies.

Parkinson’s disease (PD) is the most common neurodegenerative movement disorder affecting about 6 million people worldwide with numbers expected to rise and no cure. While the aetiology of most PD cases is poorly understood (e.g. idiopathic), rarer monogenetic forms have advanced our understanding of PD and the development of disease-modifying treatments that are currently being evaluated in clinical trials^1^. One such example is the leucine-rich repeat kinase 2 (LRRK2) and small molecule LRRK2 kinase inhibitors^1^. LRRK2 encodes a large multidomain protein, but only 7 PD-associated variants have so far been recognized as clearly disease causing^2^. The consensus is that LRRK2 mutations result in gain-of-kinase function, which is mirrored by increased LRRK2-dependent substrate phosphorylation, including Rab10 at threonine 73^2^. However, LRRK2 kinase activation may have much broader application as an underlying disease mechanism; for example, in carriers of additional LRRK2 variants of unknown clinical significance^3^, in idiopathic PD^4^ and carriers of the PD causing VPS35 D620N mutation^5^. As such, there is a need for robust and accessible markers for mechanistic disease stratification: urine mass spectrometry studies have revealed a lysosomal dysregulation signature^6^ and elevated bis(monoacylglycero)phosphate (BMP) levels^7^ in carriers of the common LRRK2 G2019S mutation. The latter is consistent with previous observations of decreased BMPs in urine of *lrrk2* knockout mice, and non-human primates treated with LRRK2 kinase inhibitors^8^. It has also been reported that treatment with the LRRK2 inhibitor DNL201 resulted in a marked decrease in urine BMP levels in 122 healthy volunteers and in 28 iPD patients in phase 1 and phase 1b clinical trials, respectively^9^. BMPs belong to a broad group of atypical, negatively charged phospholipids important for membrane formation and function in the endosome-lysosome compartment, including intraluminal vesicles and extracellularly released exosomes^10^.

Here, we have expanded urine BMP analysis to other PD-associated mutations that significantly hyperactivate the LRRK2 kinase with greater effect than LRRK2 G2019S such as R1441 hotspot mutations in LRRK2 and the VPS35 D620N mutation that activates LRRK2 kinase activity by a yet unknown mechanism.

Specifically, we included 18 heterozygous carriers of the LRRK2 G2019S mutation (11 PD/7 non-manifesting carriers (NMC)), 13 with LRRK2 R1441G/C (7 PD/6 NMC) and 10 with the pathogenic VPS35 D620N mutation (9 PD/1 NMC), as well as 10 individuals with PD associated with various GBA risk variants. In addition, we recruited one participant with atypical young onset PD and a novel homozygous ATP13A2 G38D mutation, 31 individuals with iPD and 22 healthy controls. Table 1 gives an overview of participants’ demographics, clinical status with regards to PD and genotype according to groups.

**Table 1:** Demographic information and urine BMP levels in study participants.

	Control	iPD	LRRK2 G2019S	LRRK2 R1441G/C	VPS35 D620N	GBA	*p*-value
	*N* = 22	*N* = 31	*N* = 18	*N* = 13	*N* = 10	*N* = 10	
**Age**	55	65.5	59.5	65	62.5	61	<0.001^a^
Median (range)	(24, 71)	(45, 88)	(42, 81)	(50, 83)	(29, 76)	(49, 64)	
**Sex**	11/11	15/16	11/7	6/7	7/3	3/7	0.5^b^
Female/male							
**PD status**	--	--	11/7	7/6	9/1	10/0	--
PD/NMC							
**Disease duration**	--	6	6	9	15	5	0.2^a^
Median (range)		(1, 17)	(1, 22)	(6, 20)	(1, 21)	(1, 21)	
**Total di-18:1-BMP**	3.66	6.48*	13.43****	9.57**	15.97****	2.56	<0.001^a^
Median (range)	(0.72, 8.54)	(1.48, 38.88)	(3.30, 42.56)	(2.70, 34.68)	(6.71, 55.37)	(0.80, 45.53)	
**2,2’-di-18:1-BMP**	2.04	4.58**	11.59****	4.68**	13.69****	1.58 (0.59, 21.59)	<0.001^a^
Median (range)	(0.52, 5.60)	(1.01, 34.58)	(2.86, 33.59)	(1.55, 29.59)	(4.54, 46.10)		
**Total 22:6-BMP**	8.33	12.84	43.52****	28.11***	29.19***	9.66 (3.97, 34.21)	<0.001^a^
Median (range)	(2.18, 41.14)	(3.28, 104.92)	(6.98, 321.98)	(7.94, 81.34)	(15.51, 162.36)		
**2,2’-di-22:6-BMP**	4.83	9.86*	33.43****	21.27***	22.61***	5.93 (2.81, 21.31)	<0.001^a^
Median (range)	(1.47, 19.59)	(2.17, 60.15)	(5.61, 153.74)	(4.35, 47.35)	(8.98, 126.35)		

Values are median of the measured BMP normalized to the creatinine amount (ng/mg creatinine).

GBA group includes carriers of the following variants: c.762-1 G > C, het.del-from exon3, p.Pro138Leufs*62, p.Thr408Met, p.Ser212* and p.Thr408Met, p.Glu365Lys, p.Asn409Ser.

*NMC* non-manifesting carrier.

Values significantly different from Control group: *p < 0.05; **p < 0.01; ***p < 0.001, ****p < 0.0001, Dunn’s multiple comparison test.

^a^Kruskal–Wallis rank sum test.

^b^Fisher’s exact test.

Table 1 and Fig. 1 show the data for the total and 2,2’- isoforms of the di-18:1-BMP and di-22:6-BMP species that had previously been reported to most strongly discriminate between LRRK2 G2019S mutation carriers and non-carriers^7^. All these BMP isoforms were significantly raised in the LRRK2 G2019S mutation carrier group when compared to controls. A similarly significant increase in BMP levels compared to controls was also seen in the VPS35 D620N and LRRK2 R1441G/C mutation carrier groups (Fig. 1). No statistically significant difference compared to controls was observed for the heterogeneous GBA risk variant group. With regards to iPD compared to controls, there was no significant difference for the total di-22:6 BMP isoform and an only moderate increase was observed for the other isoforms. Data for all other BMP isoforms (Supplementary Table 1 and Supplementary Fig. 1) and all possible group comparisons (Supplementary Table 2) is provided. Although there was a significant difference in age between the experimental groups (Table 1), the statistically significant differences in BMP levels were maintained when corrected for age differences (Supplementary Table 3).

**Fig. 1:**
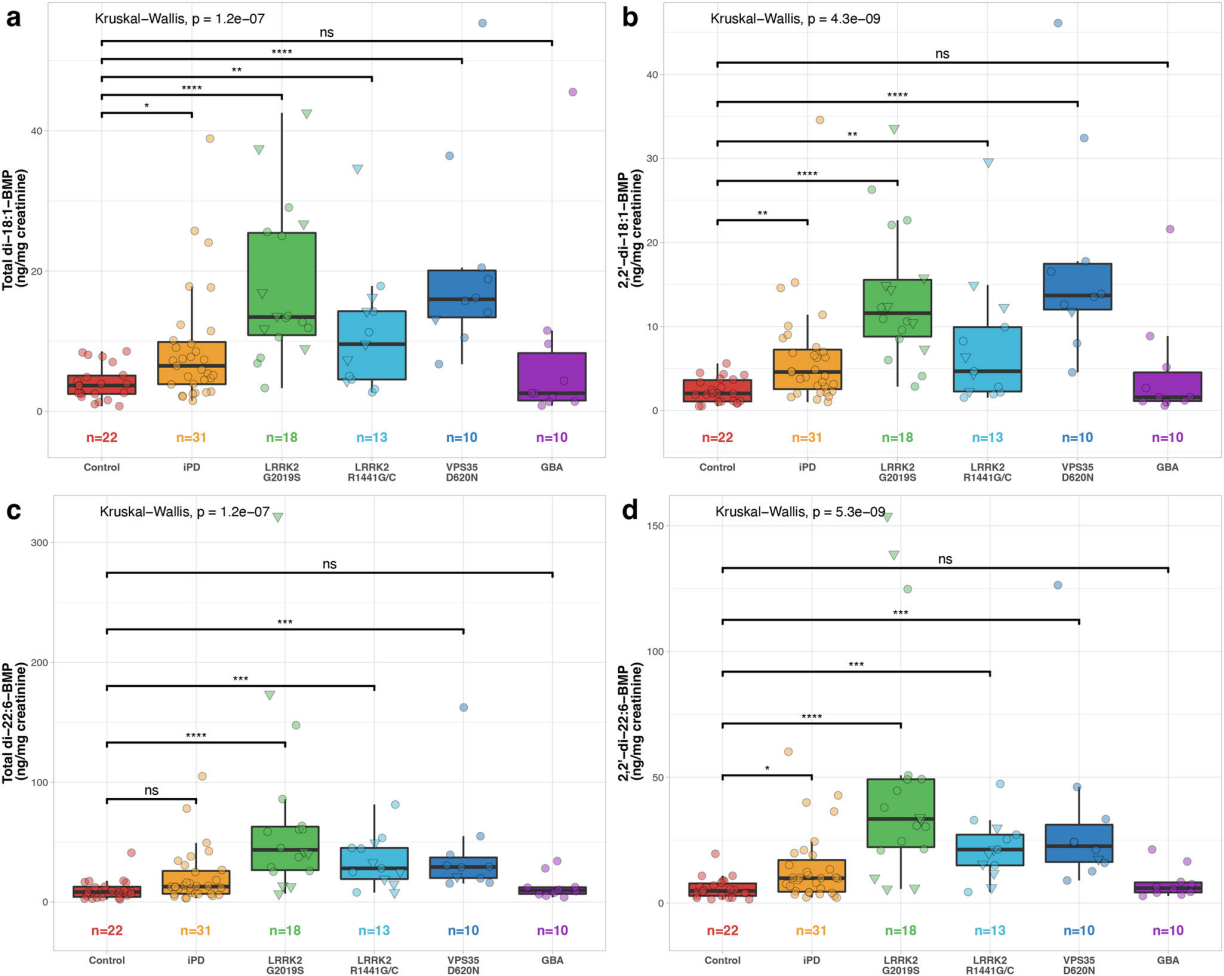
Levels of urine BMP isoforms. Urine levels of **a** 2,2’-di-18:1-BMP, **b** total di-18:1-BMP, **c** 2,2’-di-22:6-BMP, and **d** total di-22:6-BMP expressed for all groups. In the boxplots, the centre line represents the median, the bounds of the box represent the 25th and 75th percentiles, and the whiskers are set at the minimum and maximum values, excluding any outliers. Triangle datapoint shapes indicate non-manifesting mutation carriers. Statistically significant differences between groups were assessed with Kruskal–Wallis test. Post-hoc Dunn’s multiple comparison test was employed to identify groups significantly different from control (*p < 0.05, **p < 0.01, ***p < 0.001, ****p < 0.0001).

BMP levels of the ATP13A2 mutation carrier are not included in the grouped analyses, but all BMP isoforms were high, in particular the total and the 2,2’-di-18:1 isoforms (197.8, 107.7, 59.8 and 27.7 ng/mg creatinine for total-, 2,2’-di-18:1-BMP, total- and 2,2’-di-22:6-BMP, respectively), which were more than 3-fold higher than the upper range of the LRRK2 G2019S group (Table 1).

Using non-parametric Kruskal–Wallis testing, there were no significant differences between PD manifesting and non-manifesting carriers for LRRK2 G2019S and R1441 hotspot mutation carriers for any of the BMP isoforms (Supplementary Table 4). Given that there was only one non-manifesting carrier amongst the VPS35 D620N group and all GBA risk variant carriers had a diagnosis of PD, impact of clinical PD status was not assessed for these groups.

At a time of rising cases of PD and emerging disease-modifying treatments, reliable biomarkers that reflect underlying disease mechanisms and a potential for target engagement studies are urgently needed. We and others have highlighted the utility of LRRK2 substrate phosphorylation, e.g., LRRK2-dependent Rab10 phosphorylation at threonine 73, in peripheral blood as a biomarker for LRRK2 kinase activity^5,11,12^. Urine BMPs may be a complementary and in case of LRRK2 G2019S more sensitive biomarker for LRRK2 dysfunction with the additional advantage of urine being non-invasive and easier to process than peripheral blood^5,13^.

Our data together with previous results^7,9^ indicates that elevated urine BMP levels are a strong indicator of elevated LRRK2 kinase pathway activity. We observed a significant increase in all BMP isoforms measured for LRRK2 G2019S mutation carriers irrespective of PD status but did not see a significant difference between PD manifesting compared to non-manifesting LRRK2 G2019S carriers which may be due to the relatively small group size (11 PD/7 NMC). For example, the previous study was only able to observe a statistically significant increase in BMP levels in LRRK2 G2019S carriers with PD after combining the results of 2 cohorts (45 PD/36 NMC) but not separately^7^. In addition, we found a significant elevation in all BMP species measured for carriers of the LRRK2 R1441G/C as well as VPS35 D620N mutations when compared to controls. Importantly, our results provide further evidence that kinase-activating mutations in LRRK2 and the VPS35 D620N mutation operate in a shared pathway regulating endosomal-lysosomal and Golgi-sorting processes that result in elevated urine BMPs in humans^14^. While we did not observe a statistical effect of GBA variant carrier status in comparison to controls, this will need to be assessed further in much larger datasets, such as the Parkinson’s Progression Marker Initiative (PPMI, www.ppmi-info.org). The participant with ATP13A2-associated Kufor-Rabek syndrome displayed significantly elevated urine BMP levels. ATP13A2 encodes a transmembrane endolysosomal ATPase that transports polyamines into the cell regulating endolysosomal cargo-sorting and proteostasis through an interaction with the BMP precursor phosphatidylinositol^15^. A finding that contrasted with the previous study^7^ was that 3 of the 4 main urine BMP isoforms were increased in our iPD group compared to controls, albeit with relatively smaller effect size as, e.g., LRRK2 G2019S carriers. We hypothesize that this is a reflection of the heterogeneity amongst individuals with iPD in itself, which highlights the need for mechanistic stratification of PD patients.

In conclusion, we provide confirmation that urine BMP isoforms are robustly elevated in carriers of the LRRK2 G2019S mutation and provide first time evidence that urine BMPs are also elevated in LRRK2 R1441G/C and VPS35 D620N mutation carriers, consistent with shared biological pathways involved in endolysosomal trafficking. Our results also underlines that there are discriminatory biomarkers for mechanistic patient stratification that may in the future assist in matching patients with the best available targeted treatments.

## Methods

Hundred and four participants across four different sites were recruited for this study between 2019 and 2021. Controls were self-reported in general good health, did not have a diagnosis or family history of PD or tremor and did not suffer from any other neurodegenerative conditions. All participants gave written informed consent. All relevant ethics committees including the Comite Etico de Investigacion Clinica del Area Sanitaria de Gipuzkoa, Donostia Ospitalea, San Sebastian, Spain, the Comite Etico de Investigacion Clinica del Hospital Clinic de Barcelona, Spain, the Ethics Committee of the Medical University of Vienna, Austria and the North East - Newcastle & North Tyneside 2 Research Ethics Committee, United Kingdom gave ethical approval for this work.

Up to 10 mL of fresh midstream urine was centrifuged for 15 min at 2500 × *g* and 4 °C in most cases within half an hour from collection. The supernatant was transferred, aliquoted into labelled tubes, immediately snap frozen and maintained at −80 °C for storage and shipment, until analysis at Nextcea, Inc.

A multiplexed UPLC-MS/MS method was used to simultaneously quantitate molecular species of BMPs in urine, performed by Nextcea, Inc. (Woburn, MA) as before^7^. Normalization, calibration, and data processing was performed as described^7^. Sample analysis was performed blinded.

All data analysis was carried out in R (version 4.1.2) and the code has been deposited in Zenodo^16^. Non-parametric statistical tests (Kruskal–Wallis with post-hoc Dunn’s test) were used to compare the different groups defined as control, iPD, LRRK2 G2019S, LRRK2 R1441G/C, VPS35 D620N, and GBA. Adjusted *p* values are reported (*p* values lower than 0.001 are reported as *p* < 0.001), and *p* < 0.05 was considered statistically significant. To assess the potential impact of sex and age as predictors of variance of BMP levels, generalised linear models were applied.

## Supplementary information

Supplementary Figure and Tables

## Data Availability

The full BMP and clinical datasets have been deposited in Zenodo^16^.

## References

[1] Sardi SP, Cedarbaum JM, Brundin P (2018). Targeted therapies for Parkinson’s disease: from genetics to the clinic. Mov. Disord..

[2] Alessi DR, Sammler E (2018). LRRK2 kinase in Parkinson’s disease. Science.

[3] Kalogeropulou AF (2022). Impact of 100 LRRK2 variants linked to Parkinson’s disease on kinase activity and microtubule binding. Biochem. J..

[4] Di Maio R (2018). LRRK2 activation in idiopathic Parkinson’s disease. Sci. Transl. Med..

[5] Mir R (2018). The Parkinson’s disease VPS35[D620N] mutation enhances LRRK2-mediated Rab protein phosphorylation in mouse and human. Biochem. J..

[6] Virreira Winter S (2021). Urinary proteome profiling for stratifying patients with familial Parkinson’s disease. EMBO Mol. Med..

[7] Alcalay RN (2020). Higher urine bis(Monoacylglycerol)phosphate levels in LRRK2 G2019S mutation carriers: implications for therapeutic development. Mov. Disord..

[8] Fuji RN (2015). Effect of selective LRRK2 kinase inhibition on nonhuman primate lung. Sci. Transl. Med..

[9] Jennings D (2022). Preclinical and clinical evaluation of the LRRK2 inhibitor DNL201 for Parkinson’s disease. Sci. Transl. Med..

[10] Gruenberg J (2020). Life in the lumen: the multivesicular endosome. Traffic.

[11] Fan Y (2021). R1441G but not G2019S mutation enhances LRRK2 mediated Rab10 phosphorylation in human peripheral blood neutrophils. Acta Neuropathol..

[12] Wang X (2021). Understanding LRRK2 kinase activity in preclinical models and human subjects through quantitative analysis of LRRK2 and pT73 Rab10. Sci. Rep..

[13] Fan Y (2018). Interrogating Parkinson’s disease LRRK2 kinase pathway activity by assessing Rab10 phosphorylation in human neutrophils. Biochem. J..

[14] MacLeod DA (2013). RAB7L1 interacts with LRRK2 to modify intraneuronal protein sorting and Parkinson’s disease risk. Neuron.

[15] Demirsoy S (2017). ATP13A2/PARK9 regulates endo-/lysosomal cargo sorting and proteostasis through a novel PI(3, 5)P2-mediated scaffolding function. Hum. Mol. Genet..

[16] Gomes, S. et al. Elevated urine BMP phospholipids in LRRK2 and VPS35 mutation carriers with and without Parkinson’s disease. 10.5281/zenodo.7640405 (2023). 10.1038/s41531-023-00482-4 PMC10073226 37015928

