# Supplementary material for: Elevated urine BMP phospholipids in LRRK2 and VPS35 mutation carriers with and without Parkinson’s disease
Source: NPJ Parkinsons Dis. 2023 Apr 4;9:52. doi: 10.1038/s41531-023-00482-4 (PMC10073226; doi:10.1038/s41531-023-00482-4)
Supplement: Supplementary file 1 — Supplementary Figure and Tables [file 41531_2023_482_MOESM1_ESM.pdf]

Supplementary Material

Elevated urine BMP phospholipids in LRRK2 and VPS35 mutation carriers with and without Parkinson's disease

Sara Gomes PhD[1], Alicia Garrido MD[2,3], Francesca Tonelli PhD[1], Donina Obiang RN[2,3], Eduardo Tolosa MD PhD[2,3], Maria Jose Marti MD PhD[2,3,4], Javier Ruiz-Martinez MD PhD[3,5,6], Ana Vinagre Aragon MD[5,6], Haizea Hernandez-Eguiazu MSc[6], Ioana Croitoru BSc[5,6], Vicky L Marshall MD[7], Theresa Koenig PhD[8], Christoph Hotzy BSc[8], Frank Hsieh PhD[9], Marianna Sakalosh BSc[9], Elizabeth Tengstrand MSc[9], Shalini Padmanabhan PhD[10], Kalpana Merchant PhD[11], Christof Bruecke MD PhD[8], Walter Pirker MD[12], Alexander Zimprich MD[8], Esther Sammler MD PhD[1,13]

1 Medical Research Council Protein Phosphorylation and Ubiquitylation Unit, University of Dundee, Dundee DD1 5EH, UK

2 Parkinson's Disease and Movement Disorders Unit, Institut Clínic de Neurociències, Hospital Clinic Universitari, Barcelona, Spain.

3 Centre for Networked Biomedical Research on Neurodegenerative Diseases (CIBERNED), Madrid, Spain

4 Department of Clinical and Experimental Neurology, Laboratory of Parkinson disease and other Neurodegenerative Movement Disorders (IDIBAPS), University of Barcelona, Barcelona, Spain

5 Hospital Universitario Donostia, San Sebastián, Spain

6 Group of Neurodegenerative Diseases, Biodonostia Research Institute, San Sebastián, Spain

7 Neurology, Queen Elizabeth University Hospital, Institute of Neurological Sciences, Glasgow, UK

8 Department of Neurology, Medical University of Vienna, Wien, Austria

9 Nextcea, Inc. 500 West Cummings Park, Suite 4550, Woburn, Massachusetts, USA

10 The Michael J. Fox Foundation for Parkinson's Research, New York, NY, USA

11 Northwestern University Feinberg School of Medicine, Chicago, IL, USA

12 Department of Neurology, Klinik Ottakring, Vienna, Austria

13 Molecular and Clinical Medicine, Ninewells Hospital and Medical School, University of Dundee, UK

Corresponding author:

Esther Sammler MD PhD
e.m.sammler@dundee.ac.uk

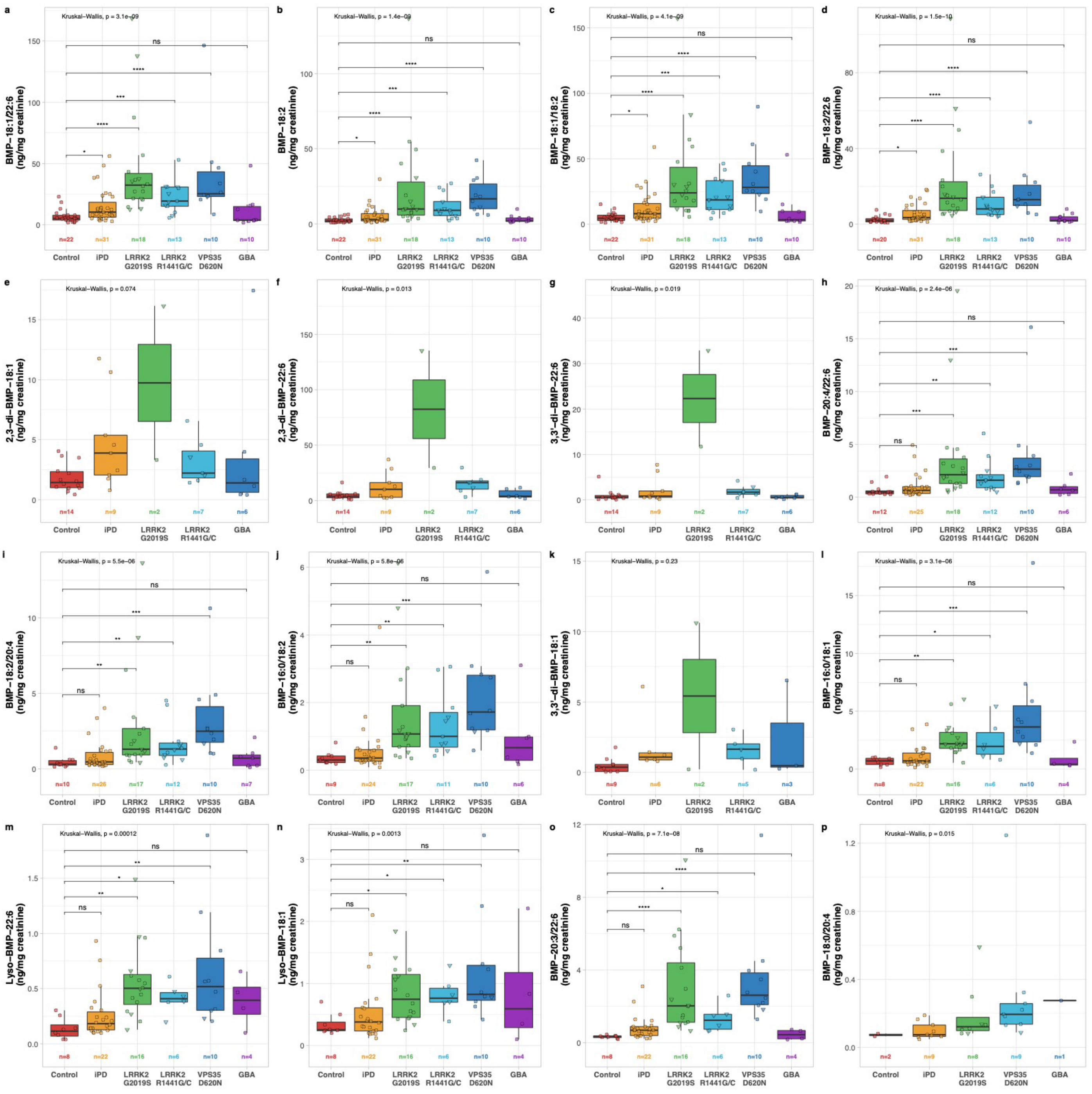

Supplementary Figure 1. Levels of urine BMP isoforms for the extended panel. Urine levels of **(a)** BMP-18:1/22:6, **(b)** BMP-18:2, **(c)** BMP-18:1/18:2, **(d)** BMP-18:2/22:6, **(e)** 2,3'-BMP-18:1, **(f)** 2,3'-BMP-22:6, **(g)** 3,3'-BMP-22:6, **(h)** BMP-20:4/22:6, **(i)** BMP-18:2/20:4, **(j)** BMP-16:0/18:2, **(k)** 3,3'-BMP-18:1, **(l)** BMP-16:0/18:1, **(m)** Lyso-BMP-22:6, **(n)** Lyso-BMP-18:1, **(o)** BMP-20:3/22:6, **(p)** BMP-18:0/20:4, expressed as ng of BMP-per mg of creatinine, are plotted per experimental group (indicated in the x-axis), in boxplots. The centre line represents the median, the bounds of the box represent the 25th and 75th percentiles, and the whiskers are set at the minimum and maximum values, excluding any outliers. Individual datapoints are shown, with triangle shapes indicating non-manifesting mutation carriers. Group size is indicated. Statistically significant differences between experimental groups were assessed with Kruskal-Wallis test, and overall p-values are displayed on the top left corner of each plot. Post-hoc Dunn's multiple comparison test was employed to identify groups significantly different from control (*$p < 0.05$, **$p < 0.01$, ***$p < 0.001$, ****$p < 0.0001$), except in cases where overall $p > 0.05$ or group size ≤ 2.

Supplementary Table 1. Levels of urine BMPs for the extended panel.

	Control		iPD		LRRK2 G2019S		LRRK2 R1441G/C		VPS35 D620N		GBA		p-value[1]
	median (range)	*N*	*median (range)*	*N*	*median (range)*	*N*	*median (range)*	*N*	*median (range)*	*N*	*median (range)*	*N*	
**BMP 18:1/22:6**	5.19 (1.86, 22.99)	22	10.38* (2.92, 56.06)	31	32.40*** (12.75, 168.50)	18	19.42*** (5.80, 53.00)	13	25.37*** (8.59, 146.41)	10	3.87 (1.74, 48.30)	10	<0.001
**BMP 18:2**	2.02 (0.72, 6.25)	22	2.97* (0.95, 29.79)	31	9.88*** (2.08, 136.82)	18	9.01*** (2.20, 27.02)	13	16.67*** (5.98, 42.34)	10	1.97 (0.95, 9.82)	10	<0.001
**BMP 18:1/18:2**	4.57 (1.14, 15.29)	22	8.07* (2.16, 58.99)	31	23.98*** (5.66, 157.33)	18	18.60*** (4.40, 46.40)	13	28.19*** (9.83, 89.80)	10	3.48 (1.63, 53.08)	10	<0.001
**BMP 18:2/22:6**	1.94 (0.77, 7.56)	20	3.51* (1.25, 18.31)	31	13.70*** (4.72, 108.74)	18	8.08*** (4.13, 26.27)	13	12.98*** (5.17, 53.91)	10	1.92 (0.89, 8.00)	10	<0.001
**2,3'-BMP 18:1[3]**	1.43 (0.43, 4.04)	14	3.88 (0.78, 11.75)	9	9.73 (3.31, 16.15)	2	2.21 (1.41, 6.56)	7	NA	0	1.39 (0.40, 17.42)	6	0.074
**2,3'-BMP 22:6[3]**	3.81 (0.96, 16.42)	14	10.15 (2.27, 37.03)	9	82.42* (29.50, 135.33)	2	16.22 (3.27, 29.80)	7	NA	0	3.93 (1.78, 11.63)	6	0.013
**3,3'-BMP 22:6[3]**	0.6 (0.08, 5.13)	14	0.84 (0.17, 7.75)	9	22.32* (11.73, 32.91)	2	1.7 (0.42, 4.19)	7	NA	0	0.53 (0.15, 1.27)	6	0.019
**BMP 20:4/22:6**	0.48 (0.27, 1.94)	12	0.65 (0.21, 4.93)	25	2.13*** (0.53, 19.57)	18	1.61** (0.52, 6.04)	12	2.66*** (1.31, 16.10)	10	0.7 (0.23, 2.21)	6	<0.001
**BMP 18:2/20:4**	0.31 (0.15, 1.40)	10	0.47 (0.19, 4.03)	26	1.31** (0.44, 13.64)	17	1.32 (0.27, 4.53)	12	2.50*** (1.02, 10.64)	10	0.72 (0.11, 2.09)	7	<0.001
**BMP 16:0/18:2**	0.3 (0.19, 0.82)	9	0.36 (0.09, 4.23)	24	1.07** (0.35, 6.13)	17	1.00** (0.42, 3.06)	11	1.72*** (0.58, 5.87)	10	0.67 (0.17, 3.10)	6	<0.001
**3,3'-BMP 18:1[3]**	0.37 (0.04, 1.79)	9	1.09 (0.80, 6.09)	6	5.42 (0.22, 10.63)	2	1.65 (0.19, 3.05)	5	NA	0	0.48 (0.27, 6.53)	3	0.2
**BMP 16:0/18:1[2]**	0.71 (0.17, 1.02)	8	0.71 (0.15, 3.87)	22	2.19** (0.55, 6.06)	16	1.96* (0.81, 5.44)	6	3.64*** (1.38, 17.82)	10	0.41 (0.30, 2.36)	4	<0.001
**Lyso-BMP 22:6[2]**	0.11 (0.04, 0.30)	8	0.18 (0.10, 0.93)	22	0.50** (0.13, 1.49)	16	0.41* (0.20, 0.61)	6	0.52** (0.21, 1.89)	10	0.39 (0.10, 0.66)	4	<0.001
**Lyso-BMP 18:1[2]**	0.25 (0.20, 0.71)	8	0.38 (0.12, 2.10)	22	0.75* (0.25, 1.85)	16	0.76* (0.39, 1.29)	6	0.83** (0.42, 3.39)	10	0.59 (0.10, 2.21)	4	0.001
**BMP 20:3/22:6[2]**	0.35 (0.20, 0.45)	8	0.69 (0.23, 3.11)	22	2.03*** (0.67, 10.07)	16	1.24* (0.66, 2.60)	6	2.62*** (1.29, 11.42)	10	0.45 (0.18, 0.72)	4	<0.001
**BMP 18:0/20:4[2]**	0.07 (0.07, 0.08)	2	0.07 (0.05, 0.19)	9	0.12 (0.08, 0.59)	8	NA	0	0.19 (0.09, 1.25)	9	0.28	1	0.015

Values are median of the measured BMP normalized to the creatinine amount (ng/mg creatinine).
[1]Kruskal-Wallis rank sum test
[2]Only measured for 2019 batch
[3]Only measured in 2021 batch
Values significantly different from Control group: *p < 0.05; **p < 0.01; ***p < 0.001, Dunn's multiple comparison test.

Supplementary Table 2. Multiple group comparison for main urine BMP isoforms. Summary of the adjusted p-values and significance level for the comparisons between the 5 experimental groups (control, iPD – idiopathic PD –, LRRK2 G2019S, LRRK2 R1441G/C, VPS35 D620N, and GBA), for the four main urine BMP species. Kruskal-Wallis test, with post-hoc Dunn's multiple comparison test (*$p < 0.05$, **$p < 0.01$, ***$p < 0.001$, ****$p < 0.0001$, ns: non-significant, $p > 0.05$).

BMP	Group 1	Group 2	N1	N2	Statistic	p	p adjusted	
Total di-18:1-BMP	Control	iPD	22	31	2.484643281	0.012968131	0.024315246	*
Total di-18:1-BMP	Control	LRRK2 G2019S	22	18	5.032357531	4.84E-07	7.27E-06	****
Total di-18:1-BMP	Control	LRRK2 R1441G/C	22	13	2.983305656	0.002851531	0.006110423	**
Total di-18:1-BMP	Control	VPS35 D620N	22	10	4.670721035	3.00E-06	2.25E-05	****
Total di-18:1-BMP	Control	GBA	22	10	0.411680876	0.680573344	0.680573344	ns
Total di-18:1-BMP	iPD	LRRK2 G2019S	31	18	3.0598777	0.002214274	0.006110423	**
Total di-18:1-BMP	iPD	LRRK2 R1441G/C	31	13	1.062241062	0.288126263	0.332453381	ns
Total di-18:1-BMP	iPD	VPS35 D620N	31	10	2.993622029	0.002756872	0.006110423	**
Total di-18:1-BMP	iPD	GBA	31	10	-1.472846748	0.140792364	0.175990455	ns
Total di-18:1-BMP	LRRK2 G2019S	LRRK2 R1441G/C	18	13	-1.526893817	0.126787417	0.172891933	ns
Total di-18:1-BMP	LRRK2 G2019S	VPS35 D620N	18	10	0.461339861	0.644554791	0.680573344	ns
Total di-18:1-BMP	LRRK2 G2019S	GBA	18	10	-3.657098977	2.55E-04	0.001054127	**
Total di-18:1-BMP	LRRK2 R1441G/C	VPS35 D620N	13	10	1.753848773	0.079456423	0.119184635	ns
Total di-18:1-BMP	LRRK2 R1441G/C	GBA	13	10	-2.107892217	0.035040314	0.058400523	ns
Total di-18:1-BMP	VPS35 D620N	GBA	10	10	-3.632121652	2.81E-04	0.001054127	**
2,2'-di-18:1-BMP	Control	iPD	22	31	3.018370144	0.002541383	0.005445821	**
2,2'-di-18:1-BMP	Control	LRRK2 G2019S	22	18	5.543878564	2.96E-08	4.44E-07	****
2,2'-di-18:1-BMP	Control	LRRK2 R1441G/C	22	13	2.792797695	0.005225436	0.009797693	**
2,2'-di-18:1-BMP	Control	VPS35 D620N	22	10	5.181977711	2.20E-07	1.65E-06	****
2,2'-di-18:1-BMP	Control	GBA	22	10	0.575248517	0.565123235	0.628681656	ns
2,2'-di-18:1-BMP	iPD	LRRK2 G2019S	31	18	3.106396608	0.001893825	0.004734563	**
2,2'-di-18:1-BMP	iPD	LRRK2 R1441G/C	31	13	0.410259653	0.681615486	0.681615486	ns
2,2'-di-18:1-BMP	iPD	VPS35 D620N	31	10	3.120655666	0.001804489	0.004734563	**
2,2'-di-18:1-BMP	iPD	GBA	31	10	-1.710435693	0.087185335	0.108981669	ns
2,2'-di-18:1-BMP	LRRK2 G2019S	LRRK2 R1441G/C	18	13	-2.156650829	0.031032881	0.046549321	*
2,2'-di-18:1-BMP	LRRK2 G2019S	VPS35 D620N	18	10	0.54352331	0.586769546	0.628681656	ns
2,2'-di-18:1-BMP	LRRK2 G2019S	GBA	18	10	-3.911126498	9.19E-05	3.44E-04	***
2,2'-di-18:1-BMP	LRRK2 R1441G/C	VPS35 D620N	13	10	2.375856092	0.01750829	0.029180483	*
2,2'-di-18:1-BMP	LRRK2 R1441G/C	GBA	13	10	-1.801140202	0.071680785	0.097746525	ns
2,2'-di-18:1-BMP	VPS35 D620N	GBA	10	10	-3.928631857	8.54E-05	3.44E-04	***
Total di-22:6-BMP	Control	iPD	22	31	1.979170939	0.047796764	0.071695146	ns
Total di-22:6-BMP	Control	LRRK2 G2019S	22	18	5.260981332	1.43E-07	2.15E-06	****
Total di-22:6-BMP	Control	LRRK2 R1441G/C	22	13	3.725156096	1.95E-04	5.86E-04	***
Total di-22:6-BMP	Control	VPS35 D620N	22	10	3.726462596	1.94E-04	5.86E-04	***
Total di-22:6-BMP	Control	GBA	22	10	0.493068842	0.621963946	0.666389942	ns
Total di-22:6-BMP	iPD	LRRK2 G2019S	31	18	3.780591867	1.56E-04	5.86E-04	***
Total di-22:6-BMP	iPD	LRRK2 R1441G/C	31	13	2.274097047	0.022960152	0.03826692	*
Total di-22:6-BMP	iPD	VPS35 D620N	31	10	2.39083987	0.016809883	0.03151853	*
Total di-22:6-BMP	iPD	GBA	31	10	-1.000030304	0.317295843	0.396619804	ns
Total di-22:6-BMP	LRRK2 G2019S	LRRK2 R1441G/C	18	13	-1.013518098	0.310812756	0.396619804	ns
Total di-22:6-BMP	LRRK2 G2019S	VPS35 D620N	18	10	-0.63597661	0.524791668	0.605528847	ns
Total di-22:6-BMP	LRRK2 G2019S	GBA	18	10	-3.762628136	1.68E-04	5.86E-04	***
Total di-22:6-BMP	LRRK2 R1441G/C	VPS35 D620N	13	10	0.280688552	0.77894929	0.77894929	ns
Total di-22:6-BMP	LRRK2 R1441G/C	GBA	13	10	-2.651082159	0.008023432	0.017193069	*
Total di-22:6-BMP	VPS35 D620N	GBA	10	10	-2.757447458	0.005825457	0.014563644	*
2,2'-di-22:6-BMP	Control	iPD	22	31	2.561885174	0.010410573	0.019519825	*
2,2'-di-22:6-BMP	Control	LRRK2 G2019S	22	18	5.827799787	5.62E-09	8.42E-08	****
2,2'-di-22:6-BMP	Control	LRRK2 R1441G/C	22	13	3.966365171	7.30E-05	2.74E-04	***
2,2'-di-22:6-BMP	Control	VPS35 D620N	22	10	4.225852833	2.38E-05	1.79E-04	***
2,2'-di-22:6-BMP	Control	GBA	22	10	0.740393438	0.4590613	0.529686115	ns
2,2'-di-22:6-BMP	iPD	LRRK2 G2019S	31	18	3.84033396	1.23E-04	3.69E-04	***
2,2'-di-22:6-BMP	iPD	LRRK2 R1441G/C	31	13	2.037849855	0.041564951	0.062347426	ns
2,2'-di-22:6-BMP	iPD	VPS35 D620N	31	10	2.467878371	0.013591649	0.022652749	*
2,2'-di-22:6-BMP	iPD	GBA	31	10	-1.187333833	0.235095968	0.29386996	ns
2,2'-di-22:6-BMP	LRRK2 G2019S	LRRK2 R1441G/C	18	13	-1.276628019	0.201733605	0.27509128	ns
2,2'-di-22:6-BMP	LRRK2 G2019S	VPS35 D620N	18	10	-0.609827792	0.541975889	0.580688453	ns
2,2'-di-22:6-BMP	LRRK2 G2019S	GBA	18	10	-3.980223657	6.89E-05	2.74E-04	***
2,2'-di-22:6-BMP	LRRK2 R1441G/C	VPS35 D620N	13	10	0.532883882	0.594113963	0.594113963	ns
2,2'-di-22:6-BMP	LRRK2 R1441G/C	GBA	13	10	-2.627438847	0.00860303	0.018435065	*
2,2'-di-22:6-BMP	VPS35 D620N	GBA	10	10	-2.97240976	0.002954721	0.007386801	**
BMP 18:1/22:6	Control	iPD	22	31	2.621690973	0.008749473	0.014582454	*
BMP 18:1/22:6	Control	LRRK2 G2019S	22	18	5.753523391	8.74E-09	1.31E-07	****
BMP 18:1/22:6	Control	LRRK2 R1441G/C	22	13	3.794404306	1.48E-04	5.55E-04	***
BMP 18:1/22:6	Control	VPS35 D620N	22	10	4.552194871	5.31E-06	3.98E-05	****
BMP 18:1/22:6	Control	GBA	22	10	0.553912273	0.579638885	0.621041663	ns
BMP 18:1/22:6	iPD	LRRK2 G2019S	31	18	3.704410676	2.12E-04	6.36E-04	***

BMP	Group 1	Group 2	N1	N2	Statistic	p	p adjusted	
BMP 18:1/22:6	iPD	LRRK2 R1441G/C	31	13	1.805336813	0.071021992	0.106532989	ns
BMP 18:1/22:6	iPD	VPS35 D620N	31	10	2.764270769	0.005705018	0.012225038	*
BMP 18:1/22:6	iPD	GBA	31	10	-1.428740737	0.153078765	0.20874377	ns
BMP 18:1/22:6	LRRK2 G2019S	LRRK2 R1441G/C	18	13	-1.377045711	0.168498116	0.210622645	ns
BMP 18:1/22:6	LRRK2 G2019S	VPS35 D620N	18	10	-0.234405476	0.814670208	0.814670208	ns
BMP 18:1/22:6	LRRK2 G2019S	GBA	18	10	-4.100694997	4.12E-05	2.06E-04	***
BMP 18:1/22:6	LRRK2 R1441G/C	VPS35 D620N	13	10	0.971800755	0.33114968	0.382095785	ns
BMP 18:1/22:6	LRRK2 R1441G/C	GBA	13	10	-2.653507114	0.007966009	0.014582454	*
BMP 18:1/22:6	VPS35 D620N	GBA	10	10	-3.409746857	6.50E-04	0.00162558	**
BMP 18:2	Control	iPD	22	31	2.258677537	0.023903451	0.035855176	*
BMP 18:2	Control	LRRK2 G2019S	22	18	5.271004267	1.36E-07	2.04E-06	****
BMP 18:2	Control	LRRK2 R1441G/C	22	13	4.105203872	4.04E-05	2.02E-04	***
BMP 18:2	Control	VPS35 D620N	22	10	5.092687763	3.53E-07	2.65E-06	****
BMP 18:2	Control	GBA	22	10	0.572877987	0.56672731	0.56672731	ns
BMP 18:2	iPD	LRRK2 G2019S	31	18	3.528401439	4.18E-04	8.96E-04	***
BMP 18:2	iPD	LRRK2 R1441G/C	31	13	2.440646624	0.014660993	0.024434988	*
BMP 18:2	iPD	VPS35 D620N	31	10	3.609351578	3.07E-04	7.67E-04	***
BMP 18:2	iPD	GBA	31	10	-1.130587114	0.258228914	0.322786142	ns
BMP 18:2	LRRK2 G2019S	LRRK2 R1441G/C	18	13	-0.656998123	0.511182123	0.547695132	ns
BMP 18:2	LRRK2 G2019S	VPS35 D620N	18	10	0.677069415	0.498361913	0.547695132	ns
BMP 18:2	LRRK2 G2019S	GBA	18	10	-3.693530397	2.21E-04	6.63E-04	***
BMP 18:2	LRRK2 R1441G/C	VPS35 D620N	13	10	1.203387176	0.228826528	0.312036175	ns
BMP 18:2	LRRK2 R1441G/C	GBA	13	10	-2.894797867	0.003794028	0.007113802	**
BMP 18:2	VPS35 D620N	GBA	10	10	-3.854506728	1.16E-04	4.35E-04	***
BMP 18:1/18:2	Control	iPD	22	31	2.295822638	0.021686024	0.036143374	*
BMP 18:1/18:2	Control	LRRK2 G2019S	22	18	5.215179036	1.84E-07	2.75E-06	****
BMP 18:1/18:2	Control	LRRK2 R1441G/C	22	13	3.881565104	1.04E-04	4.76E-04	***
BMP 18:1/18:2	Control	VPS35 D620N	22	10	4.945728119	7.59E-07	5.69E-06	****
BMP 18:1/18:2	Control	GBA	22	10	0.451982183	0.651281818	0.651281818	ns
BMP 18:1/18:2	iPD	LRRK2 G2019S	31	18	3.433584809	5.96E-04	0.001308912	**
BMP 18:1/18:2	iPD	LRRK2 R1441G/C	31	13	2.17253977	0.029814973	0.04472246	*
BMP 18:1/18:2	iPD	VPS35 D620N	31	10	3.426761343	6.11E-04	0.001308912	**
BMP 18:1/18:2	iPD	GBA	31	10	-1.285844118	0.19849747	0.261334492	ns
BMP 18:1/18:2	LRRK2 G2019S	LRRK2 R1441G/C	18	13	-0.823195934	0.410396581	0.473534516	ns
BMP 18:1/18:2	LRRK2 G2019S	VPS35 D620N	18	10	0.579946522	0.561950682	0.602090016	ns
BMP 18:1/18:2	LRRK2 G2019S	GBA	18	10	-3.765449881	1.66E-04	4.99E-04	***
BMP 18:1/18:2	LRRK2 R1441G/C	VPS35 D620N	13	10	1.256133441	0.209067594	0.261334492	ns
BMP 18:1/18:2	LRRK2 R1441G/C	GBA	13	10	-2.818419095	0.004826077	0.009048895	**
BMP 18:1/18:2	VPS35 D620N	GBA	10	10	-3.83227941	1.27E-04	4.76E-04	***
BMP 18:2/22:6	Control	iPD	20	31	2.576454944	0.009981921	0.016636536	*
BMP 18:2/22:6	Control	LRRK2 G2019S	20	18	5.810289244	6.24E-09	9.35E-08	****
BMP 18:2/22:6	Control	LRRK2 R1441G/C	20	13	4.286395287	1.82E-05	6.81E-05	****
BMP 18:2/22:6	Control	VPS35 D620N	20	10	4.921589636	8.58E-07	6.44E-06	****
BMP 18:2/22:6	Control	GBA	20	10	0.453763583	0.649998997	0.696427497	ns
BMP 18:2/22:6	iPD	LRRK2 G2019S	31	18	3.876639615	1.06E-04	2.73E-04	***
BMP 18:2/22:6	iPD	LRRK2 R1441G/C	31	13	2.38522034	0.017068895	0.025603342	*
BMP 18:2/22:6	iPD	VPS35 D620N	31	10	3.209418773	0.001330036	0.002493818	**
BMP 18:2/22:6	iPD	GBA	31	10	-1.548650979	0.121465644	0.165634969	ns
BMP 18:2/22:6	LRRK2 G2019S	LRRK2 R1441G/C	18	13	-0.990835804	0.321765763	0.402207204	ns
BMP 18:2/22:6	LRRK2 G2019S	VPS35 D620N	18	10	0.046653339	0.96278952	0.96278952	ns
BMP 18:2/22:6	LRRK2 G2019S	GBA	18	10	-4.340664749	1.42E-05	6.81E-05	****
BMP 18:2/22:6	LRRK2 R1441G/C	VPS35 D620N	13	10	0.901143293	0.367512137	0.424052466	ns
BMP 18:2/22:6	LRRK2 R1441G/C	GBA	13	10	-3.212717995	0.001314853	0.002493818	**
BMP 18:2/22:6	VPS35 D620N	GBA	10	10	-3.869250861	1.09E-04	2.73E-04	***
BMP 20:4/22:6	Control	iPD	12	25	1.030919239	0.302578694	0.41260731	ns
BMP 20:4/22:6	Control	LRRK2 G2019S	12	18	3.989054313	6.63E-05	4.98E-04	***
BMP 20:4/22:6	Control	LRRK2 R1441G/C	12	12	2.917427667	0.003529315	0.008823287	**
BMP 20:4/22:6	Control	VPS35 D620N	12	10	4.153512179	3.27E-05	4.91E-04	***
BMP 20:4/22:6	Control	GBA	12	6	0.518592826	0.604044713	0.647190764	ns
BMP 20:4/22:6	iPD	LRRK2 G2019S	25	18	3.638016262	2.75E-04	0.001030298	**
BMP 20:4/22:6	iPD	LRRK2 R1441G/C	25	12	2.360521535	0.018249259	0.03421736	*
BMP 20:4/22:6	iPD	VPS35 D620N	25	10	3.785436721	1.53E-04	7.67E-04	***
BMP 20:4/22:6	iPD	GBA	25	6	-0.226021022	0.821185071	0.821185071	ns
BMP 20:4/22:6	LRRK2 G2019S	LRRK2 R1441G/C	18	12	-0.793172427	0.42767737	0.530076993	ns
BMP 20:4/22:6	LRRK2 G2019S	VPS35 D620N	18	10	0.739835096	0.459400061	0.530076993	ns
BMP 20:4/22:6	LRRK2 G2019S	GBA	18	6	-2.603573579	0.009225746	0.019769456	*
BMP 20:4/22:6	LRRK2 R1441G/C	VPS35 D620N	12	10	1.371854042	0.17010888	0.25516332	ns
BMP 20:4/22:6	LRRK2 R1441G/C	GBA	12	6	-1.863476889	0.062395201	0.103992002	ns
BMP 20:4/22:6	VPS35 D620N	GBA	10	6	-2.94178502	0.003263264	0.008823287	**
BMP 18:2/20:4	Control	iPD	10	26	1.153617148	0.248657165	0.310821456	ns
BMP 18:2/20:4	Control	LRRK2 G2019S	10	17	3.533371884	4.10E-04	0.002051474	**
BMP 18:2/20:4	Control	LRRK2 R1441G/C	10	12	2.963264969	0.003043944	0.00760986	**

BMP	Group 1	Group 2	N1	N2	Statistic	p	p adjusted	
BMP 18:2/20:4	Control	VPS35 D620N	10	10	4.253318008	2.11E-05	3.16E-04	***
BMP 18:2/20:4	Control	GBA	10	7	0.65851919	0.51020457	0.588697581	ns
BMP 18:2/20:4	iPD	LRRK2 G2019S	26	17	3.138377187	0.001698861	0.005096583	**
BMP 18:2/20:4	iPD	LRRK2 R1441G/C	26	12	2.405586211	0.016146542	0.030274766	*
BMP 18:2/20:4	iPD	VPS35 D620N	26	10	3.958234908	7.55E-05	5.66E-04	***
BMP 18:2/20:4	iPD	GBA	26	7	-0.245984555	0.805694188	0.805694188	ns
BMP 18:2/20:4	LRRK2 G2019S	LRRK2 R1441G/C	17	12	-0.36958917	0.711688622	0.762523523	ns
BMP 18:2/20:4	LRRK2 G2019S	VPS35 D620N	17	10	1.239562141	0.215137391	0.293369169	ns
BMP 18:2/20:4	LRRK2 G2019S	GBA	17	7	-2.41293357	0.015824704	0.030274766	*
BMP 18:2/20:4	LRRK2 R1441G/C	VPS35 D620N	12	10	1.479180804	0.139091999	0.208637998	ns
BMP 18:2/20:4	LRRK2 R1441G/C	GBA	12	7	-1.985455534	0.047093813	0.078489688	ns
BMP 18:2/20:4	VPS35 D620N	GBA	10	7	-3.201304009	0.001368071	0.005096583	**
BMP 16:0/18:2	Control	iPD	9	24	0.925991025	0.354450644	0.408981513	ns
BMP 16:0/18:2	Control	LRRK2 G2019S	9	17	3.43228145	5.99E-04	0.00299263	**
BMP 16:0/18:2	Control	LRRK2 R1441G/C	9	11	3.189421724	0.001425577	0.004276732	**
BMP 16:0/18:2	Control	VPS35 D620N	9	10	4.143254183	3.42E-05	3.15E-04	***
BMP 16:0/18:2	Control	GBA	9	6	1.375822508	0.168876591	0.253314887	ns
BMP 16:0/18:2	iPD	LRRK2 G2019S	24	17	3.321600375	8.95E-04	0.003356354	**
BMP 16:0/18:2	iPD	LRRK2 R1441G/C	24	11	2.943065471	0.003249797	0.008124492	**
BMP 16:0/18:2	iPD	VPS35 D620N	24	10	4.096200027	4.20E-05	3.15E-04	***
BMP 16:0/18:2	iPD	GBA	24	6	0.795691652	0.42621128	0.456654943	ns
BMP 16:0/18:2	LRRK2 G2019S	LRRK2 R1441G/C	17	11	0.048183195	0.961570243	0.961570243	ns
BMP 16:0/18:2	LRRK2 G2019S	VPS35 D620N	17	10	1.226517814	0.220003876	0.300005285	ns
BMP 16:0/18:2	LRRK2 G2019S	GBA	17	6	-1.452584941	0.14633903	0.253314887	ns
BMP 16:0/18:2	LRRK2 R1441G/C	VPS35 D620N	11	10	1.076039422	0.281909626	0.352387033	ns
BMP 16:0/18:2	LRRK2 R1441G/C	GBA	11	6	-1.395841382	0.162762266	0.253314887	ns
BMP 16:0/18:2	VPS35 D620N	GBA	10	6	-2.28229446	0.022471961	0.048154203	*
BMP 16:0/18:1	Control	iPD	8	22	0.749887263	0.453322606	0.566653258	ns
BMP 16:0/18:1	Control	LRRK2 G2019S	8	16	3.376035279	7.35E-04	0.002757693	**
BMP 16:0/18:1	Control	LRRK2 R1441G/C	8	6	2.391352391	0.016786432	0.035970927	*
BMP 16:0/18:1	Control	VPS35 D620N	8	10	4.033218952	5.50E-05	4.13E-04	***
BMP 16:0/18:1	Control	GBA	8	4	0.031900456	0.974551435	0.974551435	ns
BMP 16:0/18:1	iPD	LRRK2 G2019S	22	16	3.506973815	4.53E-04	0.002266169	**
BMP 16:0/18:1	iPD	LRRK2 R1441G/C	22	6	2.131894203	0.033015545	0.061904147	ns
BMP 16:0/18:1	iPD	VPS35 D620N	22	10	4.204475961	2.62E-05	3.93E-04	***
BMP 16:0/18:1	iPD	GBA	22	4	-0.533641165	0.593589822	0.684911333	ns
BMP 16:0/18:1	LRRK2 G2019S	LRRK2 R1441G/C	16	6	-0.355929468	0.721893387	0.7734572	ns
BMP 16:0/18:1	LRRK2 G2019S	VPS35 D620N	16	10	1.119432261	0.262955774	0.358576056	ns
BMP 16:0/18:1	LRRK2 G2019S	GBA	16	4	-2.580120484	0.009876585	0.024691462	*
BMP 16:0/18:1	LRRK2 R1441G/C	VPS35 D620N	6	10	1.203811976	0.22866226	0.342993389	ns
BMP 16:0/18:1	LRRK2 R1441G/C	GBA	6	4	-1.970485526	0.048782752	0.081304587	ns
BMP 16:0/18:1	VPS35 D620N	GBA	10	4	-3.20074921	0.001370708	0.004112123	**
Lyso-BMP 22:6	Control	iPD	8	22	1.452655621	0.146319395	0.274348865	ns
Lyso-BMP 22:6	Control	LRRK2 G2019S	8	16	3.906655562	9.36E-05	0.001403736	**
Lyso-BMP 22:6	Control	LRRK2 R1441G/C	8	6	2.705207686	0.00682617	0.020478509	*
Lyso-BMP 22:6	Control	VPS35 D620N	8	10	3.605401748	3.12E-04	0.002337528	**
Lyso-BMP 22:6	Control	GBA	8	4	1.956826817	0.050367833	0.125919583	ns
Lyso-BMP 22:6	iPD	LRRK2 G2019S	22	16	3.323202781	8.90E-04	0.00444951	**
Lyso-BMP 22:6	iPD	LRRK2 R1441G/C	22	6	1.869945522	0.061491383	0.13176725	ns
Lyso-BMP 22:6	iPD	VPS35 D620N	22	10	2.911615967	0.003595644	0.013483665	*
Lyso-BMP 22:6	iPD	GBA	22	4	1.101192728	0.270812786	0.451354643	ns
Lyso-BMP 22:6	LRRK2 G2019S	LRRK2 R1441G/C	16	6	-0.481817048	0.629935918	0.726849136	ns
Lyso-BMP 22:6	LRRK2 G2019S	VPS35 D620N	16	10	0.046043505	0.963275574	0.963275574	ns
Lyso-BMP 22:6	LRRK2 G2019S	GBA	16	4	-0.882486009	0.377514043	0.52759737	ns
Lyso-BMP 22:6	LRRK2 R1441G/C	VPS35 D620N	6	10	0.482599058	0.629380448	0.726849136	ns
Lyso-BMP 22:6	LRRK2 R1441G/C	GBA	6	4	-0.406930216	0.684059243	0.732920617	ns
Lyso-BMP 22:6	VPS35 D620N	GBA	10	4	-0.865244258	0.386904738	0.52759737	ns
Lyso-BMP 18:1	Control	iPD	8	22	0.888269326	0.374395896	0.544135411	ns
Lyso-BMP 18:1	Control	LRRK2 G2019S	8	16	2.744495769	0.006060394	0.030301968	*
Lyso-BMP 18:1	Control	LRRK2 R1441G/C	8	6	2.433603529	0.01494936	0.044848079	*
Lyso-BMP 18:1	Control	VPS35 D620N	8	10	3.405930942	6.59E-04	0.007938872	**
Lyso-BMP 18:1	Control	GBA	8	4	1.32389655	0.185537404	0.347882633	ns
Lyso-BMP 18:1	iPD	LRRK2 G2019S	22	16	2.500786679	0.012391779	0.044848079	*
Lyso-BMP 18:1	iPD	LRRK2 R1441G/C	22	6	2.057389403	0.039648784	0.099121959	ns
Lyso-BMP 18:1	iPD	VPS35 D620N	22	10	3.274493372	0.001058516	0.007938872	**
Lyso-BMP 18:1	iPD	GBA	22	4	0.816814753	0.4140343	0.544135411	ns
Lyso-BMP 18:1	LRRK2 G2019S	LRRK2 R1441G/C	16	6	0.262985224	0.792561968	0.792561968	ns
Lyso-BMP 18:1	LRRK2 G2019S	VPS35 D620N	16	10	1.059686715	0.289287149	0.482145248	ns
Lyso-BMP 18:1	LRRK2 G2019S	GBA	16	4	-0.675621273	0.499281132	0.576093613	ns
Lyso-BMP 18:1	LRRK2 R1441G/C	VPS35 D620N	6	10	0.583423849	0.559608007	0.599580007	ns
Lyso-BMP 18:1	LRRK2 R1441G/C	GBA	6	4	-0.780140248	0.435308328	0.544135411	ns
Lyso-BMP 18:1	VPS35 D620N	GBA	10	4	-1.36045688	0.173685389	0.347882633	ns

BMP	Group 1	Group 2	N1	N2	Statistic	p	p adjusted	
BMP 20:3/22:6	Control	iPD	8	22	1.967199474	0.049160219	0.09217541	ns
BMP 20:3/22:6	Control	LRRK2 G2019S	8	16	4.63170987	3.63E-06	2.72E-05	****
BMP 20:3/22:6	Control	LRRK2 R1441G/C	8	6	2.668668887	0.007615248	0.016318389	*
BMP 20:3/22:6	Control	VPS35 D620N	8	10	4.865121841	1.14E-06	1.72E-05	****
BMP 20:3/22:6	Control	GBA	8	4	0.340271528	0.733652057	0.733652057	ns
BMP 20:3/22:6	iPD	LRRK2 G2019S	22	16	3.632190707	2.81E-04	0.001053845	**
BMP 20:3/22:6	iPD	LRRK2 R1441G/C	22	6	1.365851833	0.171985474	0.234525646	ns
BMP 20:3/22:6	iPD	VPS35 D620N	22	10	3.92136299	8.80E-05	4.40E-04	***
BMP 20:3/22:6	iPD	GBA	22	4	-1.110844875	0.266635127	0.307655916	ns
BMP 20:3/22:6	LRRK2 G2019S	LRRK2 R1441G/C	16	6	-1.17887467	0.238448085	0.298060107	ns
BMP 20:3/22:6	LRRK2 G2019S	VPS35 D620N	16	10	0.749518859	0.453544536	0.485940574	ns
BMP 20:3/22:6	LRRK2 G2019S	GBA	16	4	-3.214958255	0.001304635	0.003261587	**
BMP 20:3/22:6	LRRK2 R1441G/C	VPS35 D620N	6	10	1.67793904	0.093359	0.1400385	ns
BMP 20:3/22:6	LRRK2 R1441G/C	GBA	6	4	-1.909958666	0.056138535	0.093564226	ns
BMP 20:3/22:6	VPS35 D620N	GBA	10	4	-3.548561022	3.87E-04	0.001162027	**

Supplementary Table 3. Summary of linear models computed for each of the 4 main BMP species. Log-transformed BMP data for each of the 4 BMP species was used as the response variable, with group, age, and sex were as covariates. Each model was validated by checking residuals behaviour. LRRK2 G2019S, LRRK2 R1441G/C, and VPS35 D620N groups remain significantly different from control (p <0.05), even when age and sex are included in the linear model.

	Total di-18:1-BMP		2,2'-di-18:1-BMP		Total di-22:6-BMP		2,2'-di-22:6-BMP	
	Estimate	p-value	Estimate	p-value	Estimate	p-value	Estimate	p-value
**(Intercept)**	0.696	0.065	0.195	0.606	0.651	0.076	0.160	0.653
**iPD**	0.474	0.051	0.682**	0.006	0.150	0.525	0.266	0.248
**LRRK2 G2019S**	1.339***	<0.001	1.652***	<0.001	1.349***	<0.001	1.539***	<0.001
**LRRK2 R1441G/C**	0.716*	0.018	0.785*	0.010	0.733*	0.013	0.818**	0.005
**VPS35 D620N**	1.581***	<0.001	1.965***	<0.001	1.086***	<0.001	1.347***	<0.001
**GBA**	-0.066	0.828	0.033	0.913	0.016	0.957	0.044	0.879
**Age**	0.013	0.067	0.012	0.082	0.029***	<0.001	0.029***	<0.001
**Sex**	-0.242	0.121	-0.279	0.077	0.029	0.850	-0.017	0.906

*p<0.05; **p<0.01; ***p<0.001

Supplementary Table 4. Comparison between PD-manifesting and non-manifesting LRRK2 mutation carriers for main urine BMP isoforms. Summary of the adjusted p-values and significance level for the comparison between PD-manifesting (PD) and non-manifesting (NMC) carriers of LRRK2 G2019S or LRRK2 R1441G/C mutations, for the four main urine BMP species. Kruskal-Wallis test, with post-hoc Dunn's multiple comparison test (ns: non-significant, *p* > 0.05).

BMP	Group 1	Group 2	N1	N2	Statistic	p-value
Total di-18:1-BMP	PD	NMC	27	14	0.726	0.394
2,2'-di-18:1-BMP	PD	NMC	27	14	0.823	0.364
Total di-22:6-BMP	PD	NMC	27	14	3.096	0.078
2,2'-di-22:6-BMP	PD	NMC	27	14	2.631	0.105
BMP 18:1/22:6	PD	NMC	27	14	0.333	0.564
BMP 18:2	PD	NMC	27	14	0.003	0.956
BMP 18:1/18:2	PD	NMC	27	14	0.170	0.680
BMP 18:2/22:6	PD	NMC	27	14	0.926	0.336
2,3'-BMP 18:1	PD	NMC	6	3	0.600	0.439
2,3'-BMP 22:6	PD	NMC	6	3	0.267	0.606
3,3'-BMP 22:6	PD	NMC	6	3	0.600	0.439
BMP 20:4/22:6	PD	NMC	26	14	2.895	0.089
BMP 18:2/20:4	PD	NMC	25	14	1.238	0.266
BMP 16:0/18:2	PD	NMC	25	13	0.173	0.678
3,3'-BMP 18:1	PD	NMC	5	2	1.350	0.245
BMP 16:0/18:1	PD	NMC	21	11	0.067	0.796
Lyso-BMP 22:6	PD	NMC	21	11	0.662	0.416
Lyso-BMP 18:1	PD	NMC	21	11	0.429	0.513
BMP 20:3/22:6	PD	NMC	21	11	5.205	0.023
BMP 18:0/20:4	PD	NMC	13	4	0.542	0.461
